# Dissecting the Genetic Regulation of Yeast Growth Plasticity in Response to Environmental Changes

**DOI:** 10.3390/genes11111279

**Published:** 2020-10-29

**Authors:** Yanjun Zan, Örjan Carlborg

**Affiliations:** 1 Department of Forestry Genetics and Plant Physiology, Swedish University of Agricultural Sciences, 90736 Umeå, Sweden; 2 Department of Medical Biochemistry and Microbiology, Uppsala University, 75123 Uppsala, Sweden; orjan.carlborg@imbim.uu.se; 3 National Key Laboratory of Crop Genetic Improvement, Huazhong Agricultural University, Wuhan 430070, China

**Keywords:** yeast growth, genotype by environment interactions, epistasis, genetic networks, phenotype plasticity

## Abstract

Variable individual responses to environmental changes, such as phenotype plasticity, are heritable, with some genotypes being robust and others plastic. This variation for plasticity contributes to variance in complex traits as genotype-by-environment interactions (G × E). However, the genetic basis of this variability in responses to the same external stimuli is still largely unknown. In an earlier study of a large haploid segregant yeast population, genotype-by-genotype-by-environment interactions were found to make important contributions to the release of genetic variation in growth responses to alterations of the growth medium. Here, we explore the genetic basis for heritable variation of different measures of phenotype plasticity in the same dataset. We found that the central loci in the environmentally dependent epistatic networks were associated with overall measures of plasticity, while the specific measures of plasticity identified a more diverse set of loci. Based on this, a rapid one-dimensional genome-wide association (GWA) approach to overall plasticity is proposed as a strategy to efficiently identify key epistatic loci contributing to the phenotype plasticity. The study thus provided both analytical strategies and a deeper understanding of the complex genetic regulation of phenotype plasticity in yeast growth.

## 1. Introduction

The ability of individuals to change their phenotypes according to the surrounding environments is termed as phenotype plasticity. It is known to be genetically regulated with a moderate heritability and could respond to selection in artificial selection experiments [1,2,3,4,5,6,7]. With the availability of large-scale genome-wide markers and phenotype data in the past thirty years, many studies have acknowledged phenotype plasticity as an intriguing factor resulting in gene-by-environment interactions for complex traits impacting evolution and adaptation, and challenging the definition of genotype-phenotype maps [8,9]. Recently, it has been shown that gene-by-gene interactions (epistasis) can be dependent on environment, where such non-additive effects can contribute to the emergence of extreme phenotypes [10] and likely also facilitate rapid responses to artificial selection by the induction of selectable genetic variation [11,12,13]. The genetic basis for gene-by-environment interactions could, due to the close link between the two concepts, be dissected either by mapping loci independently in different environments and exploring changes in effects, or by mapping loci affecting phenotype plasticity directly. Although both approaches have been used, there are few empirical studies that have compared the two approaches in the same data to explore how the associated genetic polymorphisms, and their interactions, overlap [14].

Earlier studies dissecting the genetic variation in yeast growth have revealed contributions from environment-specific quantitative trait loci (QTLs) with alleles displaying genotype-by-genotype (G-by-G) and genotype-by-environment interactions (G-by-E) [4,5,6,7,15,16,17]. Here, we continue the analyses of an experimental yeast dataset where we earlier identified extensive genetic epistatic interaction networks. These influenced yeast growth and had variable effects across the multiple evaluated growth media (G-by-G-by-E interactions) [18,19]. A remaining question from that study was the potential link between the changes in the genetic interaction networks across environments (G-by-G-by-E) and the phenotype plasticity of individual segregants (genotypes). Here, this question was approached by quantifying the phenotype plasticity of growth measures for individual haploid segregants (genotypes) across multiple environments, and associating the variation in several measures of overall and specific plasticity to genetic polymorphisms in this large intercross population [20]. Many overall phenotype plasticity QTL were also central loci (hubs) in the earlier identified interaction networks explaining growth variation across different media. This illustrates an important connection between overall phenotype plasticity and genotype-by-genotype-by-environment interactions mediated by interaction networks and how the key epistatic loci could be identified using single-locus association mapping targeting phenotype plasticity loci.

## 2. Materials and Methods

### 2.1. Data

We analyzed a public dataset with 4390 haploid segregants from a cross between a laboratory strain (BY) and an isolate from a vineyard (RM). Every segregant strain was genotyped for ~30 K single nucleotide polymorphisms (SNPs) and phenotyped for colony size on 20 mediums with various chemical additives, including Cobalt Chloride, Copper Sulphate, Diamide, E6-Berbamine, Ethanol, Formamide, Hydroxyurea, Indole-acetic acid, Lactate, lactose Magnesium Chloride, Manganese Sulphate, Menadione Neomycin, Raffinose, Trehalose, Xylose, and Zeocin. The details on the population development, genotyping, phenotyping, and data pre-processing are available in Bloom et al. [20]. Growth data from 2 of the 20 media (YPD and YNB) were excluded from the analyses here since they were used as control media to normalize the environmental effect for the remaining 18 media. Previously detected additive QTL, epistatic QTLs, as well as within-environment interaction networks, were downloaded from the supplementary data of Forsberg et al. [19] and Zan et al. [18].

### 2.2. Quantifying Yeast Growth Plasticity

In this study, the phenotype of each haploid segregant (genotype) was scored as the average of multiple normalized replicated measurements of colony size. This was used as a measure of growth and repeatedly performed on the 20 growth media (here defined as environments) 48 h after plating [20]. Since the same segregants (genotypes) were scored across all environments, this allowed us to quantify and study the genetics of this growth plasticity in response to a range of chemical environmental perturbations.

**Figure 1:**
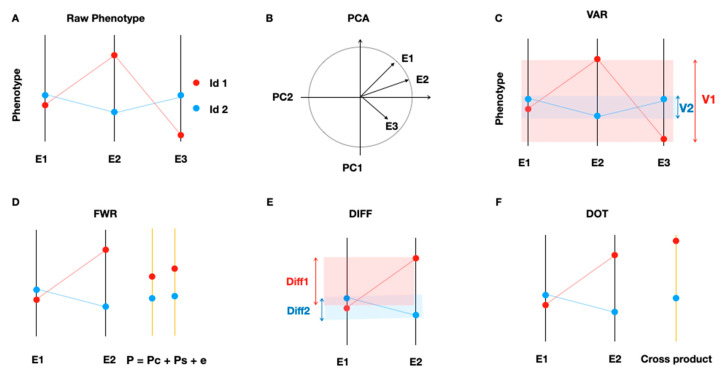
Schematic illustrations of the five approaches evaluated to quantify the phenotype plasticity. (**A**) Illustration of the concept of phenotypic plasticity. Each bar represents an environment and the average phenotype of two haploid segregants (genotypes, id1 and id2) are illustrated using red/blue dots. Average growths for the segregants (horizontal blue and red lines) vary across three environments (vertical black lines). The growth for one segregant (red) varies more (is more plastic) in response to the environment than the other (blue). Three measures of overall phenotype (growth) plasticity across all environments (growth media) are used and schematically illustrated in (**B**–**D**). The first, *PCA*, uses a principle component analysis of a matrix containing growth measures of segregants in all media. (**B**) A variable loading plot showing the contributions by the growth in three media (environments) to the first two principal components (PC1 and PC2). The two PCs explaining the most variance are used as measures of plasticity (abbreviated *PCA1* and *PCA2*). The second overall measure (*VAR,* (**C**)) quantifies plasticity as the across environment variance of individual segregant growth. The third overall measurement (*FWR*, (**D**)) partitions growth into two components, one (*Pc*) that is constant across environments and another (Ps) that varies across environments. Here, growths (phenotypes) measured on two media (environments, E1 and E2) are illustrated on the left, resulting in a hypothetical partitioning in the two components on the right. Two specific growth plasticity measures for each of the pairs of media (environments) are used. (**E**) *DIFF* that is quantified as the pairwise growth difference, and (**F**) *DOT* quantified as the cross product of standardized growth measurements between paired media (environments, E1 and E2).

In this study, we used 5 metrics to quantify the growth plasticity that are schematically illustrated in Figure 1B–E. Three measures (abbreviated as PCA, VAR, and FWR, see below) were considered overall measures of plasticity since they score the plasticity across all evaluated environments. Another two measures (abbreviated DIFF and DOT) were considered specific measures of plasticity since they scored the plasticity separately for each pair of environments.

The principle component analysis (PCA) approach was the first overall score of plasticity. The basis for this approach is to perform a PCA analysis of the phenotype matrix, i.e., the *n* × *e* matrix, where each row represents a segregant (genotype) and each column represent the growth of these segregants on a given medium (environment). The influence of the growth measures in each environment on the principle components (PCs) can be illustrated using a loading plot (Figure 1B) [21]. Multiple PCs can capture variation in growth across the media where, for example, the loading on the second PC was negative for medium (environment) 3 and positive for media (environments) 1 and 2 (Figure 1B). This means that both the first and second PCs capture variation in overall plasticity, and therefore they were used as measures of the overall phenotypic (growth) plasticity (*PCA1* and *PCA2*).

The second overall score of plasticity is the across environment variance (*VAR*) proposed in Reference [22] (Figure 1C). This was calculated as the across environment variance of the growth measurements.

The third score for the overall plasticity (FWR) uses a Finlay–Wilkinson Regression (Figure 1D) [23,24]. This approach assumes that the growth (phenotype) of an individual in a particular environment is composed by two components, one which is constant across environments and another one which is plastic and responds to environmental perturbation [23,24]. Using linear regression, the phenotype of each strain is partitioned into these two components and the plasticity component is used here in the downstream analyses.

In addition to the three measures of overall plasticity of growth across all media, two measures of specific plasticity defined for each pair of media are included. The first one is a quantification of the pairwise growth differences (DIFF), where for the individuals, the differences in colony size were calculated independently for all possible pairs of environments [25,26] (Figure 1E). This resulted in a total of (18 × 17)/2 = 154 specific DIFF scores.

The second measure of specific plasticity was the dot product between the standardized individual growth phenotypes measured for the segregants in two environments (Figure 1F). This was an estimate of the phenotypic correlation for the growths on two media estimated as the pairwise correlation, *r,* between two environments (*r* = $\frac{\sum^{\;}\left(X-\overset{=}{X}\right)\left(Y-\overset{=}{Y}\right)}{\sqrt{\sum^{\;}{\left(X-\overset{=}{X}\right)}^{2}}\sqrt{\sum^{\;}{\left(Y-\overset{=}{Y}\right)}^{2}}}$) which is the same as the dot product when the phenotype is standardized ($\overset{=}{X}=\overset{=}{Y}=0$ resulting in $\sqrt{\sum^{\;}{\left(X-\overset{=}{X}\right)}^{2}}=\sqrt{\sum^{\;}{\left(Y-\overset{=}{Y}\right)}^{2}}$ = 1, therefore, satisfying r = $\sum^{\;}XY$). This resulted in a total of (18 × 17)/2 = 154 specific DOT scores. Thereafter, the genetic basis of variation in these six estimates of growth plasticity, calculated using these five methods, were explored using association analysis.

### 2.3. Heritability Estimation

To estimate the narrow sense heritability of each plasticity measurement, a linear mixed model was fitted:

(1) Y= μ + Zu + e

where *Y* is a vector of the growth (phenotype) plasticity measurement, assumed to be normally distributed with mean 0. μ  is the population mean and *Z* is the design matrix obtained from a Cholesky decomposition of the kinship matrix *G* estimated using the *ibs* function (option weight = ‘freq’) in the *GenABEL* package [27]. The *Z* matrix satisfies *ZZ*′ = *G*, therefore, the random effect *u* is normally distributed (*u*~*N* (0, *I*$\sigma_{g}^{2}$)). *e* is the residual variance with *e* ~ *N* (0, $I\sigma_{e}^{2}$). The narrow sense heritability of fitted phenotype was calculated as the intraclass correlation $\sigma_{g}^{2}$/($\sigma_{g}^{2}\;$+ $\sigma_{e}^{2}$), and this was implemented in the *polygenic* function in the *GenABEL* package [27].

### 2.4. Mapping Alleles Underlying Variation in Phenotypic Plasticity

To map genetic polymorphisms associated with variation in growth plasticity, we fitted a second linear mixed model (2):

(2) Y= μ + Xβ + Zu +e

where *Y*, μ, Z, *u,* and *e* are defined as in (1). *X* is a matrix containing the genotype of the tested SNP(s) (coded as 0/2 for minor/major-allele homozygous genotypes, respectively). *β* is a vector including the estimated additive allele-substitution effect for the tested SNP(s). First, a genome-wide analysis (GWA) across all genotyped SNPs was conducted using the R package *GenABEL* [27]. Next, a subsequent conditional analysis was performed where all the top associated SNPs (the SNPs with the highest *p*-value) from each association peak from the initial GWA scan were included as covariates in the linear mixed model (LMM) to screen for additional association signals. This conditional analysis was implemented in the *cojo* test in the software *GCTA* [28].

### 2.5. Estimating the Significance Threshold for the Association Analyses

The level of linkage disequilibrium (LD) was extensive in this intercross, making a Bonferroni-corrected significance threshold that assumes all tested markers to be independent over-conservative. We, therefore, estimated the effective number of independent markers (*Me*) from the eigenvalues of the marker correlation matrix [29]. Based on this, the genome-wide significance threshold was estimated as 0.05/Me (1.93 × 10^−5^).

### 2.6. Connecting Plasticity QTL to Previously Detected Epistatic Networks

In our previous analysis of this dataset [18,19], 11 epistatic networks with 13 hubs were detected underlying the variation of growth means. These epistatic networks were highly environmental-dependent, with the radial loci being connected or disconnected in response to changing the growth medium. To evaluate the overlap between plasticity QTL identified in this study, and QTL–QTL interaction pairs from earlier reported exhaustive two-locus interaction analyses across all possible combinations of genome-wide polymorphisms, we downloaded the results from References [18,19,20]. As LD is extensive in this intercross population, overlapping of plasticity and interaction QTL were defined if they met both criteria: (i) physical distance within 50 kb and (ii) LD (r^2^) > 0.5.

## 3. Results

### 3.1. Heritable Variation in Yeast Growth Plasticity in Response to Chemical Stress

**Figure 2:**
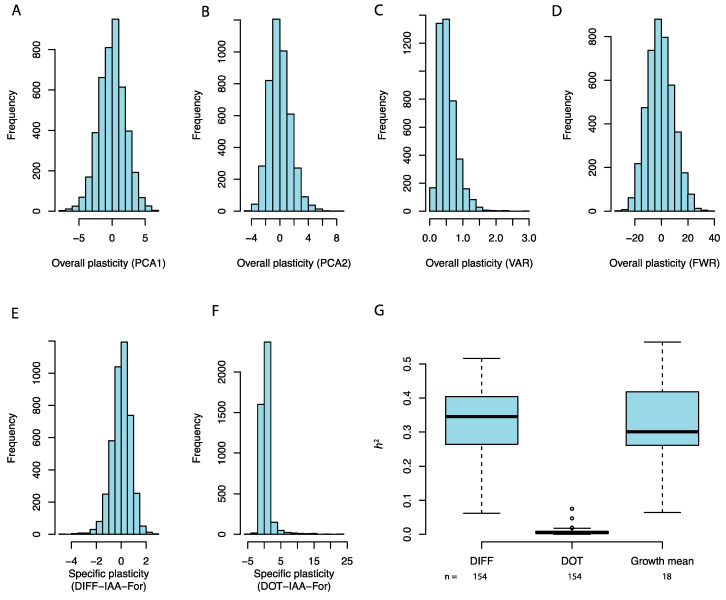
Distributions and narrow sense heritabilities of the evaluated plasticity measurements. The distributions of the overall plasticity measures (First principle component, PCA1 and second principle component, PCA2, (**A**,**B**)), within environment variance (VAR, (**C**)), and the Finlay–Wilkinson plastic component (FWR, (**D**)). The distribution of the specific plasticity of growth between media with added Indole Acetic Acid (IAA) and Formamide measured as growth differences (DIFF, (**E**)) and dot product (DOT, (**F**)). (**G**) Boxplots of the estimated narrow sense heritabilities for plasticity measurements DIFF, DOT, and raw growth mean, respectively.

The distribution of the overall measures of plasticity are presented in Figure 2 as the two first principle components of the phenotype matrix (PCA1 and PCA2; Figure 2A,B), the within strain variance across the 18 media (VAR; Figure 2C), and the stability parameter from the Finlay–Wilkinson regression (FWR; Figure 2D). In total, there are 308 measurements for the specific plasticity, 154 DIFF measures and 154 DOT measurements, with each of these measures representing the plasticity measurements for a pairwise combination of growth environments. Figure 2E, F present the distributions for the DIFF (E) and DOT (F) estimates between the growth media with added Indole Acetic Acid and Formamide. All plasticity measures display a continuous variation (Figure 2A–F). The distributions for the kinship heritabilities of growth in the 18 media (Figure 2G) was similar to the distribution in heritabilities for the specific plasticity measure DIFF (Figure 2G) with a median around 0.3, in contrast to the lower estimates obtained for DOT (Figure 2C; median around 0). For the overall plasticity measurements, the estimates were 0.41, 0.35, 0.05, and 0.66 for PCA1, PCA2, VAR, and FWR, respectively.

### 3.2. Mapping Alleles Contributing to the Variation in Growth Plasticity

Genome-wide association analyses (GWA) were performed for (i) growth on the 18 different media, (ii) the overall growth plasticity measures (PCA1 and PCA2, VAR, and FWR), and (iii) the 308 specific plasticity measures (Number of measurements (n) are nDIFF = 154 and nDOT = 154). At a genome-wide significance level (*p*-value = 1.93 × 10^−5^), 36 independent QTLs were detected for the 18 trait means, 11 independent QTLs for the overall plasticity measurements, and 52 independent QTLs for the 308 environmental-specific plasticity measurements (Supplementary Tables S1 and S2).

**Figure 3:**
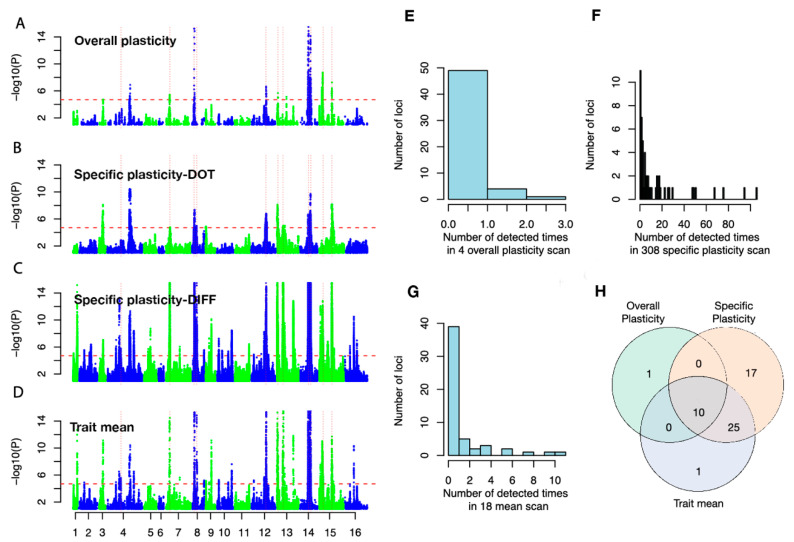
Summary of results from the association analyses of the plasticity measurements. (**A**–**D**) Manhattan plots overlaying the genome-wide association scans for the 4 overall plasticity measures ((**A**), PCA1/PCA2/VAR/FWR), 154 specific plasticity measurements quantified as the pairwise dot product ((**B**), DOT), the 154 specific plasticity quantified as pairwise difference ((**C**), DIFF), and 18 trait means (**D**), respectively. The red horizontal dashed lines in (**A**–**D**) indicate the Bonferroni-corrected genome-wide significance threshold derived as 0.05/Me (Me is the effective number of independent single nucleotide polymorphisms, SNPs; Materials and Methods Section). SNPs with *p*-values below this threshold in (**A**–**D**) were declared to be statistically significant. (**E**–**G**) The total number of times a QTL is detected in the overall plasticity, pairwise plasticity, and trait mean GWA scans. (**H**) The overlap between QTL detected for overall plasticity, pairwise plasticity, and trait means.

After evaluating the overlap in locations of the QTL (Figure 3A–D), there were 54 unique QTL in total. The majority of the QTL were only detected for one of the GWA scans, while a few QTL were repeatedly detected in many scans (Figure 3E–G). Most of the QTL (10 of 11; Figure 3H) associated with variation in the measures of overall plasticity (FWR, VAR, PCA1, or PCA2) were also mapped in the GWA for growth mean in at least one environment and at least one of the specific plasticity measures (DIFF or DOT). In total, 17 QTL were unique to the specific plasticity measurements (Figure 3H).

### 3.3. Many Plasticity QTLs are Hub Loci in Previously Detected Epistatic Networks

**Figure 4:**
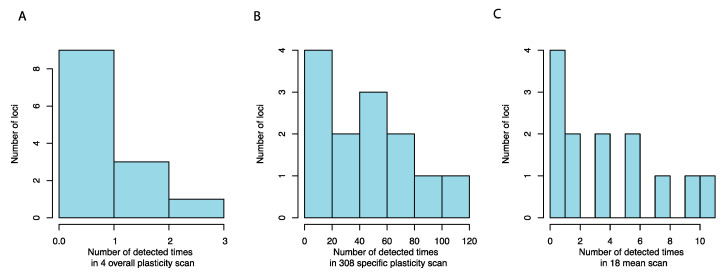
The detection frequency for hub loci in mean and plasticity GWA scan. The number of times the 13 previously detected epistatic hub-loci in growth networks of this population [19] were mapped as plasticity QTL in GWA scans for overall plasticity (**A**), pairwise plasticity (**B**), and growth (**C**) traits.

In total, 9 out of the 11 QTL mapped for the overall plasticity measures (FWR, VAR, PCA1, PCA2) overlapped with hubs in the 11 epistatic networks (containing 13 hubs in total) that contributed to growth in this population [18,19]. All of these 13 epistatic network hubs were mapped for at least one of the specific plasticity measurements, GWA, with 11/13 using the DOT/DIFF measurements, respectively (Figure 3A–D and Figure 4A–C).

### 3.4. A Known Epistatic Growth Hub QTL Affecting Specific and Overall Plasticity

**Figure 5:**
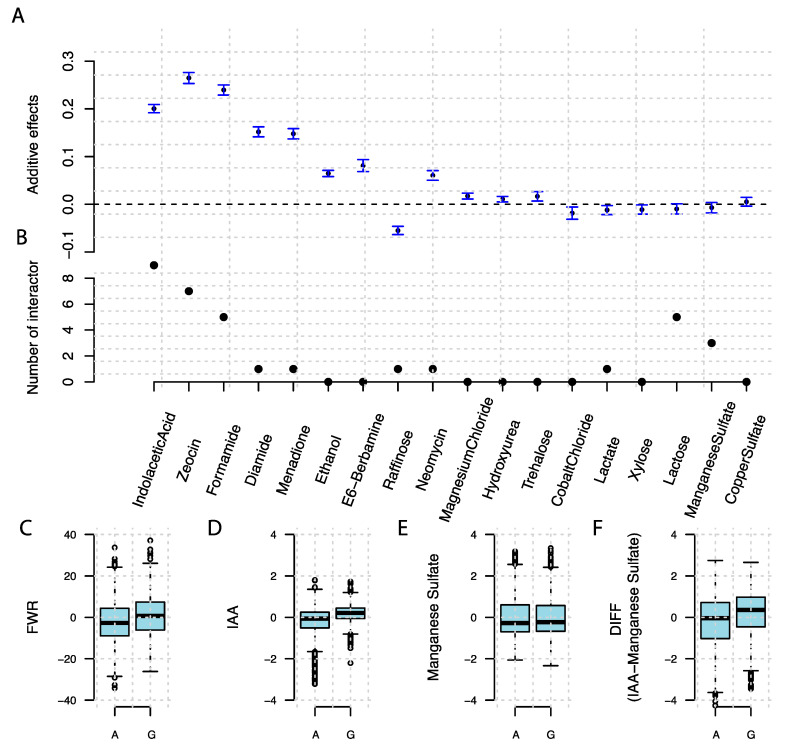
Genetic effects of the epistatic hub locus chromosome 8 (117,586 bp) on trait means, and specific and overall plasticity. (**A**) Marginal additive effects on growth in the 18 media. (**B**) The number of reported epistatic interactors for this hub in the earlier genome-wide exhaustive pairwise epistatic scan [20]. (**C**) Genotype-to-phenotype map for overall plasticity (FWR) measured as the stability parameter of the Finlay–Wilkinson Regression estimated at this QTL, (**D**–**F**) Genotype-to-phenotype maps for growth measure and on media with added IAA (**D**) or Manganese Sulphate (**E**), as well as the specific plasticity quantified by their pairwise growth differences for the segregants (DIFF, (**F**)). Two genotypes were present at this locus, A and G, and the boxplot illustrates the phenotype distributions for these.

A known hub locus in an epistatic network on chromosome 8 (117,586 bp) [19] was associated to growth on media with added Indole Acetic Acid (IAA), Zeocin, Formamide, and Diamide. It was also associated with 51 specific plasticity measurements (47 DIFF and 4 DOT) and two overall plasticly measurements (FWD and PCA2). Both the additive effects and the number of epistatic interactors of this hub locus varied across the 18 media (Figure 5A,B) and were significantly correlated (R = 0.64, *p* = 0.004). Two genotypes were present at this locus for these haploid sergeants, one with genotype A and the second with G. On media where it interacted with more loci, the G genotype has a larger effect on colony size, and when the epistatic network breaks down, this marginal additive effect fades away. This variation in the allelic effect across environments results in a larger overall growth plasticity for individuals with the G genotype as measured by FWR (Figure 5C). As is shown in Figure 5D,E, the G genotype has a significant positive additive effect on colony size on the medium containing IAA, but not on the medium with added Manganese Sulphate. This results in the G genotype having a larger difference in growth between these two mediums, resulting in a significant association between this locus and the pairwise phenotypic difference. The segregants with genotype G are thus more plastic than segregants with the A genotype across these growth environments.

## 4. Discussion

Studying the genetic basis of phenotype plasticity requires data and analyses to explore changes in the genotype-to-phenotype map across multiple environments. The most powerful data will thus include multiple observations for each genotype in every environment. For inbred or clones, repeated measures can be collected for the same individual in multiple environments. For many species, including outbred animals or annual outbred plants, collecting such data from many individuals across multiple environments is generally not feasible. However, in perennial or inbred plants, such studies are possible but time-consuming and costly, and association mapping in such study design will be challenging due to the unbalanced allele frequencies and population structure. Experimental crosses of segregating inbred or haplotype individuals are a useful experimental design for this purpose, and large-scale studies in terms of both numbers of individuals and environments are possible in microorganisms. Here, we used yeast as a model to explore the genetic basis of phenotype plasticity. The flexible mating system facilitated the generation of large haploid segregant populations from crosses of diverse populations, and its ability to rapidly proliferate in a range of environments makes it a powerful model system to investigate the genetic regulation of phenotypic plasticity.

### 4.1. Quantification of the Phenotypic Plasticity

Studies on phenotypic plasticity require phenotypic measurements for individuals in two or more environments. Phenotype plasticly is, however, not explicitly defined and will be affected both by the data—traits measured and combination of environments studied—as well as the statistical method used to quantify it. The dataset studied included >4000 haploid individuals with replicated growth measures in 20 growth environments. This allowed us to evaluate how robust the detection of growth plasticity loci were to both the choice of environment and measures of plasticity in a study. To this end, we used three methods (four measures) to quantify overall growth plasticity across all studied environments and two methods (308 measures) to quantify specific plasticity for pairs of environments. In this way, a comprehensive evaluation is provided to understand the role of individual QTL in the variation of overall and specific plasticity as well as whether some earlier proposed plasticity measures are likely to be more useful in genetic mapping studies.

Our results show that, even though the overall plasticity measurements were correlated, different QTLs were often detected, suggesting that they capture different aspects of plasticity and that they could provide complementary information in future studies. When comparing QTL detected for overall and specific plasticity, most of the QTLs (>90%) detected for overall plasticity were also detected for specific plasticity, while many more QTLs were detected for specific plasticity. This is expected since these pairwise measures will put a stronger emphasis on individual differences in phenotypes between pairs of environments than the overall measures. In future studies, it is therefore important to select overall or specific plasticity measures that are of greatest relevance for the research question as QTLs with different types of effects will be detected. An intriguing finding was that most of the known hub loci in epistatic networks contributing to growth were associated with the overall plasticity measurements. This suggests that the dynamics of the underlying epistatic networks in response to environmental perturbations [18,19] are also an important contributor to variation of overall phenotype plasticity of individuals.

### 4.2. The Role of QTL–QTL and QTL–Environment Interactions in Phenotype Plasticity

Earlier studies exploring the contributions by individual, and combinations of, loci on mean growth with and across growth media have revealed extensive QTL–QTL interactions and QTL–environment interactions in this population [18,19,20]. Here, this work was extended to also map the loci contributing to variation in growth of individuals across media defined as one trait, growth plasticity. Most of the key loci-hubs—in the epistatic networks involved in QTL-by-QTL and QTL-by-environments interactions—overlapped with QTL, regulating overall growth plasticity. This suggests that, from a statistical perspective, the overall plasticity measures used here capture the same variance in growth as the scans for genetic and genotype-by-environment interactions. Since this one-dimensional QTL scan reduces computing cost considerably compared to the higher-dimensional scans for interactions, it is a promising approach to identify key loci to be involved in such network interactions.

### 4.3. Relationship to Earlier Studies Evaluating the Role of G-by-G-by-E Interactions on Growth Variation in Individual Environments

Previously, the growth measurements from different media have been analyzed as independent traits using this dataset [19,20]. The later study from Forsberg et al. revealed multiple epistatic networks in several environments, suggesting that the non-additive genetic component played a very important role in variation of the growth within each environment. In a more recent study by Zan et al. [18], the epistatic growth networks were found to result in the emergence of extreme phenotypes, and release of genetic variation, after environmental perturbations. Unexplored was, however, how the dynamics in the networks was connected to the phenotype plasticity that likely drives the emerging G-by-G-by-E interactions. Instead of focusing on traits/genetic architecture mapped in individual environments and making post hoc comparisons, here, we extended the earlier work by directly quantifying plasticity using multiple measurements and performing genome-wide QTL scans on these plasticity measurements. The large overlap between the epistatic hubs, detected in earlier studies [18,19], that respond dynamically to environmental perturbation [18] and QTLs associated with the overall growth plasticity, demonstrated that variation in overall growth plasticity was related to the dynamics in the underlying epistatic networks. Overall, these results suggest that a polygenic genetic architecture underlying the variation of growth plasticity with a few loci have effects on both trait mean and plasticity, with many additional loci then uniquely affecting specific plasticity measurements. Most of the overlapping loci were epistatic hubs that were earlier shown to respond dynamically to environmental changes. Analogous to our results, Bhatia et al. [16] found two locus epistatic interactions that were conditional on environment and contributed to the variation of growth plasticity assayed using liquid media in another yeast cross including 150 segregants. The overlaps in the molecular pathways contributing to growth measured in liquid and on solid media are largely unknown. These high-level, complex phenotypes result from the actions and interactions of many different cell biological and physiological traits. Differences in demands on division rates, cell sizes and shapes, cell–cell interactions, and more, during growth in liquid and on solid media, make large overlaps unlikely. However, despite the fact that there are likely differences in which molecular mechanisms contribute to growth, the agreement in findings between our study and that of Bhatia et al. [16] suggests that rewiring of epistatic interaction networks is an important feature regardless of this difference in underlying pathways. To the best of our knowledge, findings from the yeast population analyzed here provide a comprehensive empirical example illustrating how non-additive interactions shape the within and across environment phenotype variation. Further, it also shows how variation in the underlying genetic architecture for individual environments alters the amount of genetic variation as well as phenotype plasticity, resulting in some individuals being more/less robust than others. Based on this first study, it is too early to make definitive conclusions about whether this is a generally important mechanism in yeast or underlying phenotype plasticity in general. However, it suggests that it is the time to start experimentally evaluating whether the findings from this particular cross also hold in other populations of yeast as well as other species.

### 4.4. Candidate Genes Contributing to Variation in Phenotype Plasticity

Linkage disequilibrium extends too far to directly pinpoint the genes underlying the mapped QTL. A manual screen for genes in the associated regions identified a number functional candidates, including *GPA1* [30], *HAP1* [31,32,33], *KRE33* [34], *MKT1* [35], and *IRA2* [36], all located in the epistatic hubs and whose phenotypic effects have been explored in previous studies of this population [18,19]. Future experimental validation is needed to define how they might contribute to epistasis, genotype-by-environment interactions, and phenotypic plasticity. Earlier expression QTL studies showed that several growth QTL that were hubs in the epistatic networks were also epistatic hubs for expression of many genes [37]. For example, the epistatic interaction between *HAP1* and *MKT1* and between *HAP1* and *KRE33* is related to variations in expression levels of many genes [38]. Although the current results do not provide conclusive evidence about how interactions are connected to plasticity in yeast growth, the allelic and environmental dependencies of these epistatic networks allow the formulation of new and experimentally testable hypotheses to clarify this in the future.

## 5. Conclusions

We found that both overall and specific growth plasticity are heritable in this population and the underlying loci can be mapped by associating the variation in quantified plasticity measures to the genetic polymorphisms using GWAS. Nearly all loci associated with overall plasticity overlapped with QTL for specific plasticity QTL and also with epistatic growth network hub QTL detected in individual environments. Specific plasticity QTL were more often unique to pairs of growth environments and plasticity measures. We proposed the one-dimensional overall plasticity mapping approach as a powerful and computationally efficient strategy to identify highly interactive loci in epistatic networks. These results present further insights to the genetic basis of within and across environment phenotypic variation in this population, that loci contributing to individual plasticity in responses to environmental perturbation can be mapped, and that these, to a large extent, overlap with key loci involved in high-order interaction networks regulating growth in yeast.

## Supplementary Materials

The following are available online at https://www.mdpi.com/2073-4425/11/11/1279/s1. A summary of the detected SNPs is available as supplementary Tables S1 and S2. Table S1: Summary of the 54 SNPs associated with the growth mean and plasticity measurements; Table S2: Variant effect prediction for the 54 associated SNPs.
