# Supplementary material for: Dissecting the Genetic Regulation of Yeast Growth Plasticity in Response to Environmental Changes
Source: Genes (Basel). 2020 Oct 29;11(11):1279. doi: 10.3390/genes11111279 (PMC7693874; doi:10.3390/genes11111279)
Supplement: Supplementary file 1 [file genes-11-01279-s001.pdf]

Table S2 Variant effect prediction for the 54 associated SNPs

SNPs	Chr	Pos
40866_chrI_40866_T_C	1	40866
40866_chrI_40866_T_C	1	40866
40866_chrI_40866_T_C	1	40866
40866_chrI_40866_T_C	1	40866
40866_chrI_40866_T_C	1	40866
40866_chrI_40866_T_C	1	40866
114628_chrI_114628_G_T	1	114628
114628_chrI_114628_G_T	1	114628
114628_chrI_114628_G_T	1	114628
114628_chrI_114628_G_T	1	114628
114628_chrI_114628_G_T	1	114628
194104_chrI_194104_C_T	1	194104
194104_chrI_194104_C_T	1	194104
475523_chrII_245305_C_T	2	245305
475523_chrII_245305_C_T	2	245305
475523_chrII_245305_C_T	2	245305
475523_chrII_245305_C_T	2	245305
475523_chrII_245305_C_T	2	245305
475523_chrII_245305_C_T	2	245305
475523_chrII_245305_C_T	2	245305
684479_chrII_454261_G_A	2	454261
684479_chrII_454261_G_A	2	454261
684479_chrII_454261_G_A	2	454261
684479_chrII_454261_G_A	2	454261
684479_chrII_454261_G_A	2	454261
684479_chrII_454261_G_A	2	454261
684479_chrII_454261_G_A	2	454261
684479_chrII_454261_G_A	2	454261
774352_chrII_544134_G_A	2	544134
774352_chrII_544134_G_A	2	544134
774352_chrII_544134_G_A	2	544134
774352_chrII_544134_G_A	2	544134
774352_chrII_544134_G_A	2	544134
774352_chrII_544134_G_A	2	544134
774352_chrII_544134_G_A	2	544134
1118075_chrIII_74673_A_G	3	74673
1118075_chrIII_74673_A_G	3	74673
1118075_chrIII_74673_A_G	3	74673
1118075_chrIII_74673_A_G	3	74673
1118075_chrIII_74673_A_G	3	74673
1118075_chrIII_74673_A_G	3	74673
1118075_chrIII_74673_A_G	3	74673
1248152_chrIII_204750_G_A	3	204750
1248152_chrIII_204750_G_A	3	204750

1248152_chrIII_204750_G_A	3	204750
1248152_chrIII_204750_G_A	3	204750
1248152_chrIII_204750_G_A	3	204750
1766121_chrIV_406099_C_T	4	406099
1766121_chrIV_406099_C_T	4	406099
1766121_chrIV_406099_C_T	4	406099
1766121_chrIV_406099_C_T	4	406099
1899218_chrIV_539196_C_A	4	539196
1899218_chrIV_539196_C_A	4	539196
1899218_chrIV_539196_C_A	4	539196
1899218_chrIV_539196_C_A	4	539196
1966016_chrIV_605994_T_C	4	605994
1966016_chrIV_605994_T_C	4	605994
1966016_chrIV_605994_T_C	4	605994
1966016_chrIV_605994_T_C	4	605994
1966016_chrIV_605994_T_C	4	605994
1966016_chrIV_605994_T_C	4	605994
1966016_chrIV_605994_T_C	4	605994
2358650_chrIV_998628_A_T	4	998628
2358650_chrIV_998628_A_T	4	998628
2358650_chrIV_998628_A_T	4	998628
2358650_chrIV_998628_A_T	4	998628
2358650_chrIV_998628_A_T	4	998628
2424307_chrIV_1064285_G_A	4	1064285
2424307_chrIV_1064285_G_A	4	1064285
2424307_chrIV_1064285_G_A	4	1064285
2424307_chrIV_1064285_G_A	4	1064285
2424307_chrIV_1064285_G_A	4	1064285
2518625_chrIV_1158603_T_A	4	1158603
2518625_chrIV_1158603_T_A	4	1158603
2518625_chrIV_1158603_T_A	4	1158603
3180989_chrV_289034_G_A	5	289034
3180989_chrV_289034_G_A	5	289034
3180989_chrV_289034_G_A	5	289034
3180989_chrV_289034_G_A	5	289034
3180989_chrV_289034_G_A	5	289034
3180989_chrV_289034_G_A	5	289034
3927817_chrVII_188827_A_G	7	188827
3927817_chrVII_188827_A_G	7	188827
3927817_chrVII_188827_A_G	7	188827
3927817_chrVII_188827_A_G	7	188827
3927817_chrVII_188827_A_G	7	188827
3927817_chrVII_188827_A_G	7	188827
3927817_chrVII_188827_A_G	7	188827
3927817_chrVII_188827_A_G	7	188827
4348841_chrVII_609851_G_A	7	609851

4348841_chrVII_609851_G_A	7	609851
4348841_chrVII_609851_G_A	7	609851
4348841_chrVII_609851_G_A	7	609851
4348841_chrVII_609851_G_A	7	609851
4947516_chrVIII_117586_A_G	8	117586
4947516_chrVIII_117586_A_G	8	117586
4947516_chrVIII_117586_A_G	8	117586
4947516_chrVIII_117586_A_G	8	117586
4947516_chrVIII_117586_A_G	8	117586
5040640_chrVIII_210710_A_T	8	210710
5040640_chrVIII_210710_A_T	8	210710
5040640_chrVIII_210710_A_T	8	210710
5040640_chrVIII_210710_A_T	8	210710
5040640_chrVIII_210710_A_T	8	210710
5040640_chrVIII_210710_A_T	8	210710
5040640_chrVIII_210710_A_T	8	210710
5040640_chrVIII_210710_A_T	8	210710
5352702_chrVIII_522772_A_C	8	522772
5352702_chrVIII_522772_A_C	8	522772
5352702_chrVIII_522772_A_C	8	522772
5426372_chrIX_33799_G_A	9	33799
5426372_chrIX_33799_G_A	9	33799
5556905_chrIX_164332_C_T	9	164332
5556905_chrIX_164332_C_T	9	164332
5556905_chrIX_164332_C_T	9	164332
5556905_chrIX_164332_C_T	9	164332
5652805_chrIX_260232_C_T	9	260232
5652805_chrIX_260232_C_T	9	260232
5652805_chrIX_260232_C_T	9	260232
5652805_chrIX_260232_C_T	9	260232
5652805_chrIX_260232_C_T	9	260232
5652805_chrIX_260232_C_T	9	260232
5940441_chrX_107980_A_G	10	107980
5940441_chrX_107980_A_G	10	107980
5940441_chrX_107980_A_G	10	107980
5940441_chrX_107980_A_G	10	107980
5940441_chrX_107980_A_G	10	107980
6083002_chrX_250541_T_A	10	250541
6083002_chrX_250541_T_A	10	250541
6083002_chrX_250541_T_A	10	250541
6083002_chrX_250541_T_A	10	250541
6083002_chrX_250541_T_A	10	250541
6340191_chrX_507730_A_G	10	507730
6340191_chrX_507730_A_G	10	507730
6340191_chrX_507730_A_G	10	507730
6491373_chrX_658912_C_T	10	658912

6491373_chrX_658912_C_T	10	658912
6491373_chrX_658912_C_T	10	658912
7198325_chrXI_620113_T_C	11	620113
7198325_chrXI_620113_T_C	11	620113
7198325_chrXI_620113_T_C	11	620113
7380059_chrXII_135031_G_A	12	135031
7380059_chrXII_135031_G_A	12	135031
7380059_chrXII_135031_G_A	12	135031
7380059_chrXII_135031_G_A	12	135031
7380059_chrXII_135031_G_A	12	135031
7380059_chrXII_135031_G_A	12	135031
7693396_chrXII_448368_A_G	12	448368
7693396_chrXII_448368_A_G	12	448368
7815060_chrXII_570032_G_A	12	570032
7815060_chrXII_570032_G_A	12	570032
7815060_chrXII_570032_G_A	12	570032
7815060_chrXII_570032_G_A	12	570032
7815060_chrXII_570032_G_A	12	570032
7815060_chrXII_570032_G_A	12	570032
7815060_chrXII_570032_G_A	12	570032
7902564_chrXII_657536_G_T	12	657536
7995649_chrXII_750621_C_T	12	750621
7995649_chrXII_750621_C_T	12	750621
7995649_chrXII_750621_C_T	12	750621
7995649_chrXII_750621_C_T	12	750621
8347770_chrXIII_24565_A_G	13	24565
8347770_chrXIII_24565_A_G	13	24565
8347770_chrXIII_24565_A_G	13	24565
8347770_chrXIII_24565_A_G	13	24565
8347770_chrXIII_24565_A_G	13	24565
8347770_chrXIII_24565_A_G	13	24565
8474614_chrXIII_151409_T_C	13	151409
8474614_chrXIII_151409_T_C	13	151409
8474614_chrXIII_151409_T_C	13	151409
8474614_chrXIII_151409_T_C	13	151409
8474614_chrXIII_151409_T_C	13	151409
8595700_chrXIII_272495_G_A	13	272495
8595700_chrXIII_272495_G_A	13	272495
8595700_chrXIII_272495_G_A	13	272495
8595700_chrXIII_272495_G_A	13	272495
8595700_chrXIII_272495_G_A	13	272495
8595700_chrXIII_272495_G_A	13	272495
8656654_chrXIII_333449_G_A	13	333449
8656654_chrXIII_333449_G_A	13	333449
8656654_chrXIII_333449_G_A	13	333449
8656654_chrXIII_333449_G_A	13	333449

8656654_chrXIII_333449_G_A	13	333449
8656654_chrXIII_333449_G_A	13	333449
8736983_chrXIII_413778_C_T	13	413778
8736983_chrXIII_413778_C_T	13	413778
8736983_chrXIII_413778_C_T	13	413778
8736983_chrXIII_413778_C_T	13	413778
8736983_chrXIII_413778_C_T	13	413778
8736983_chrXIII_413778_C_T	13	413778
8736983_chrXIII_413778_C_T	13	413778
9024358_chrXIII_701153_G_C	13	701153
9024358_chrXIII_701153_G_C	13	701153
9024358_chrXIII_701153_G_C	13	701153
9024358_chrXIII_701153_G_C	13	701153
9024358_chrXIII_701153_G_C	13	701153
9093436_chrXIII_770231_G_C	13	770231
9093436_chrXIII_770231_G_C	13	770231
9093436_chrXIII_770231_G_C	13	770231
9093436_chrXIII_770231_G_C	13	770231
9142223_chrXIII_819018_C_T	13	819018
9142223_chrXIII_819018_C_T	13	819018
9142223_chrXIII_819018_C_T	13	819018
9142223_chrXIII_819018_C_T	13	819018
9142223_chrXIII_819018_C_T	13	819018
9531198_chrXIV_283562_A_G	14	283562
9531198_chrXIV_283562_A_G	14	283562
9531198_chrXIV_283562_A_G	14	283562
9531198_chrXIV_283562_A_G	14	283562
9531198_chrXIV_283562_A_G	14	283562
9624071_chrXIV_376435_C_G	14	376435
9624071_chrXIV_376435_C_G	14	376435
9624071_chrXIV_376435_C_G	14	376435
9624071_chrXIV_376435_C_G	14	376435
9624071_chrXIV_376435_C_G	14	376435
9624071_chrXIV_376435_C_G	14	376435
9624071_chrXIV_376435_C_G	14	376435
9662617_chrXIV_414981_T_C	14	414981
9662617_chrXIV_414981_T_C	14	414981
9662617_chrXIV_414981_T_C	14	414981
9662617_chrXIV_414981_T_C	14	414981
9729532_chrXIV_481896_A_G	14	481896
9729532_chrXIV_481896_A_G	14	481896
9729532_chrXIV_481896_A_G	14	481896
9729532_chrXIV_481896_A_G	14	481896
9729532_chrXIV_481896_A_G	14	481896
9729532_chrXIV_481896_A_G	14	481896
9729532_chrXIV_481896_A_G	14	481896

9871041_chrXIV_623405_A_T	14	623405
9871041_chrXIV_623405_A_T	14	623405
9871041_chrXIV_623405_A_T	14	623405
9871041_chrXIV_623405_A_T	14	623405
9871041_chrXIV_623405_A_T	14	623405
9871041_chrXIV_623405_A_T	14	623405
9871041_chrXIV_623405_A_T	14	623405
10109136_chrXV_77167_C_T	15	77167
10109136_chrXV_77167_C_T	15	77167
10109136_chrXV_77167_C_T	15	77167
10109136_chrXV_77167_C_T	15	77167
10109136_chrXV_77167_C_T	15	77167
10109136_chrXV_77167_C_T	15	77167
10184891_chrXV_152922_C_T	15	152922
10184891_chrXV_152922_C_T	15	152922
10184891_chrXV_152922_C_T	15	152922
10184891_chrXV_152922_C_T	15	152922
10427207_chrXV_395238_T_C	15	395238
10427207_chrXV_395238_T_C	15	395238
10427207_chrXV_395238_T_C	15	395238
10427207_chrXV_395238_T_C	15	395238
10589592_chrXV_557623_T_C	15	557623
10589592_chrXV_557623_T_C	15	557623
10589592_chrXV_557623_T_C	15	557623
10589592_chrXV_557623_T_C	15	557623
10589592_chrXV_557623_T_C	15	557623
10589592_chrXV_557623_T_C	15	557623
10653941_chrXV_621972_A_G	15	621972
10653941_chrXV_621972_A_G	15	621972
10653941_chrXV_621972_A_G	15	621972
10653941_chrXV_621972_A_G	15	621972
11330697_chrXVI_207437_T_A	16	207437
11330697_chrXVI_207437_T_A	16	207437
11330697_chrXVI_207437_T_A	16	207437
11330697_chrXVI_207437_T_A	16	207437
11330697_chrXVI_207437_T_A	16	207437
11498453_chrXVI_375193_A_T	16	375193
11498453_chrXVI_375193_A_T	16	375193
11498453_chrXVI_375193_A_T	16	375193
11498453_chrXVI_375193_A_T	16	375193
11498453_chrXVI_375193_A_T	16	375193
11498453_chrXVI_375193_A_T	16	375193
11625348_chrXVI_502088_C_A	16	502088
11625348_chrXVI_502088_C_A	16	502088
11625348_chrXVI_502088_C_A	16	502088
11625348_chrXVI_502088_C_A	16	502088

11625348_chrXVI_502088_C_A	16	502088
11625348_chrXVI_502088_C_A	16	502088
11625348_chrXVI_502088_C_A	16	502088

Annotation	Impact	Gene
downstream_gene_variant	MODIFIER	ACS1
upstream_gene_variant	MODIFIER	PEX22
synonymous_variant	LOW	GPB2
downstream_gene_variant	MODIFIER	CNE1
downstream_gene_variant	MODIFIER	ECM1
downstream_gene_variant	MODIFIER	BDH1
downstream_gene_variant	MODIFIER	LDS1
upstream_gene_variant	MODIFIER	FUN30
upstream_gene_variant	MODIFIER	ATS1
upstream_gene_variant	MODIFIER	CCR4
upstream_gene_variant	MODIFIER	FUN26
downstream_gene_variant	MODIFIER	YAT1
missense_variant	MODERATE	SWH1
upstream_gene_variant	MODIFIER	NTH2
upstream_gene_variant	MODIFIER	RER2
downstream_gene_variant	MODIFIER	COQ1
synonymous_variant	LOW	GPI18
upstream_gene_variant	MODIFIER	RCR1
upstream_gene_variant	MODIFIER	UGA2
downstream_gene_variant	MODIFIER	DSF2
downstream_gene_variant	MODIFIER	SIF2
downstream_gene_variant	MODIFIER	YMC2
upstream_gene_variant	MODIFIER	VID24
downstream_gene_variant	MODIFIER	PHO88
synonymous_variant	LOW	IML3
upstream_gene_variant	MODIFIER	AIM3
downstream_gene_variant	MODIFIER	CMD1
upstream_gene_variant	MODIFIER	ALG1
downstream_gene_variant	MODIFIER	YSW1
downstream_gene_variant	MODIFIER	ARA1
synonymous_variant	LOW	TBS1
upstream_gene_variant	MODIFIER	APD1
upstream_gene_variant	MODIFIER	SPP381
upstream_gene_variant	MODIFIER	RIB7
downstream_gene_variant	MODIFIER	RPB5
upstream_gene_variant	MODIFIER	KCC4
downstream_gene_variant	MODIFIER	AGP1
downstream_gene_variant	MODIFIER	FRM2
upstream_gene_variant	MODIFIER	HBN1
downstream_gene_variant	MODIFIER	FUS1
downstream_gene_variant	MODIFIER	RNQ1
upstream_gene_variant	MODIFIER	BIK1
upstream_gene_variant	MODIFIER	MATALPHA2
downstream_gene_variant	MODIFIER	MATALPHA1

synonymous_variant	LOW	TAF2
downstream_gene_variant	MODIFIER	PER1
downstream_gene_variant	MODIFIER	RRT12
downstream_gene_variant	MODIFIER	DIA3
missense_variant	MODERATE	RTK1
upstream_gene_variant	MODIFIER	MRX9
upstream_gene_variant	MODIFIER	MPS1
upstream_gene_variant	MODIFIER	ENA2
upstream_gene_variant	MODIFIER	ENA1
upstream_gene_variant	MODIFIER	RSM10
downstream_gene_variant	MODIFIER	NRG1
downstream_gene_variant	MODIFIER	SED1
upstream_gene_variant	MODIFIER	SHU2
upstream_gene_variant	MODIFIER	TFB5
downstream_gene_variant	MODIFIER	PET100
missense_variant	MODERATE	VPS41
downstream_gene_variant	MODIFIER	PDC2
upstream_gene_variant	MODIFIER	STN1
upstream_gene_variant	MODIFIER	DIN7
upstream_gene_variant	MODIFIER	AKR1
upstream_gene_variant	MODIFIER	PEX10
downstream_gene_variant	MODIFIER	HEL2
downstream_gene_variant	MODIFIER	CIA1
downstream_gene_variant	MODIFIER	BFR2
upstream_gene_variant	MODIFIER	PRO1
missense_variant	MODERATE	CFT1
upstream_gene_variant	MODIFIER	GPI11
downstream_gene_variant	MODIFIER	RSC3
upstream_gene_variant	MODIFIER	HXT7
downstream_gene_variant	MODIFIER	HXT6
downstream_gene_variant	MODIFIER	HXT3
upstream_gene_variant	MODIFIER	VHR2
upstream_gene_variant	MODIFIER	ICL1
upstream_gene_variant	MODIFIER	RRT13
upstream_gene_variant	MODIFIER	RGI1
upstream_gene_variant	MODIFIER	MOT2
upstream_gene_variant	MODIFIER	SUP19
downstream_gene_variant	MODIFIER	RAD54
downstream_gene_variant	MODIFIER	YRB30
upstream_gene_variant	MODIFIER	CUP2
missense_variant	MODERATE	PMR1
downstream_gene_variant	MODIFIER	HUR1
downstream_gene_variant	MODIFIER	SUA5
upstream_gene_variant	MODIFIER	SPO74
downstream_gene_variant	MODIFIER	ROK1
upstream_gene_variant	MODIFIER	LST7

downstream_gene_variant	MODIFIER	PEF1
downstream_gene_variant	MODIFIER	SPR3
upstream_gene_variant	MODIFIER	ERG25
downstream_gene_variant	MODIFIER	ADE6
upstream_gene_variant	MODIFIER	NEM1
upstream_gene_variant	MODIFIER	TIM10
upstream_gene_variant	MODIFIER	GPA1
upstream_gene_variant	MODIFIER	STP2
downstream_gene_variant	MODIFIER	ERG11
downstream_gene_variant	MODIFIER	YHK8
downstream_gene_variant	MODIFIER	FSH1
downstream_gene_variant	MODIFIER	SMF2
downstream_gene_variant	MODIFIER	COX6
upstream_gene_variant	MODIFIER	CIC1
downstream_gene_variant	MODIFIER	CUP1-1
downstream_gene_variant	MODIFIER	CUP1-2
downstream_gene_variant	MODIFIER	RSC30
downstream_gene_variant	MODIFIER	BAT1
downstream_gene_variant	MODIFIER	CRG1
upstream_gene_variant	MODIFIER	FLO5
upstream_gene_variant	MODIFIER	SUC2
downstream_gene_variant	MODIFIER	NIT1
downstream_gene_variant	MODIFIER	SLM1
upstream_gene_variant	MODIFIER	MOB1
missense_variant	MODERATI	PFK26
upstream_gene_variant	MODIFIER	SEC24
downstream_gene_variant	MODIFIER	SYG1
upstream_gene_variant	MODIFIER	NEO1
synonymous_variant	LOW	DFG10
downstream_gene_variant	MODIFIER	PCL7
upstream_gene_variant	MODIFIER	MMF1
upstream_gene_variant	MODIFIER	RPL34B
downstream_gene_variant	MODIFIER	TPK1
synonymous_variant	LOW	HAL5
downstream_gene_variant	MODIFIER	QCR8
downstream_gene_variant	MODIFIER	ERG20
upstream_gene_variant	MODIFIER	SET2
downstream_gene_variant	MODIFIER	TOK1
downstream_gene_variant	MODIFIER	KHA1
missense_variant	MODERATI	BCK1
downstream_gene_variant	MODIFIER	MRPL49
downstream_gene_variant	MODIFIER	PHS1
upstream_gene_variant	MODIFIER	HUL4
upstream_gene_variant	MODIFIER	GEF1
downstream_gene_variant	MODIFIER	URB2
upstream_gene_variant	MODIFIER	ENT3

upstream_gene_variant	MODIFIER	VPS70
downstream_gene_variant	MODIFIER	RSF2
downstream_gene_variant	MODIFIER	PTR2
upstream_gene_variant	MODIFIER	RPL40B
synonymous_variant	LOW	MLP1
downstream_gene_variant	MODIFIER	SPO75
upstream_gene_variant	MODIFIER	MMM1
synonymous_variant	LOW	LMO1
downstream_gene_variant	MODIFIER	DRS1
upstream_gene_variant	MODIFIER	COX17
upstream_gene_variant	MODIFIER	PSR1
upstream_gene_variant	MODIFIER	ACS2
upstream_gene_variant	MODIFIER	RNH203
upstream_gene_variant	MODIFIER	TUB4
upstream_gene_variant	MODIFIER	CRR1
missense_variant	MODERATI	FRE1
downstream_gene_variant	MODIFIER	CDC123
downstream_gene_variant	MODIFIER	CPR6
downstream_gene_variant	MODIFIER	COA4
upstream_gene_variant	MODIFIER	MSC3
upstream_gene_variant	MODIFIER	GSY2
downstream_gene_variant	MODIFIER	CDA1
downstream_gene_variant	MODIFIER	CDA2
synonymous_variant	LOW	IMH1
downstream_gene_variant	MODIFIER	CDC25
downstream_gene_variant	MODIFIER	NDI1
upstream_gene_variant	MODIFIER	GTR1
synonymous_variant	LOW	PHO84
upstream_gene_variant	MODIFIER	TUB3
upstream_gene_variant	MODIFIER	PGA3
upstream_gene_variant	MODIFIER	ERG13
downstream_gene_variant	MODIFIER	NTE1
upstream_gene_variant	MODIFIER	OGG1
missense_variant	MODERATI	PIF1
upstream_gene_variant	MODIFIER	MFT1
downstream_gene_variant	MODIFIER	RPS1B
downstream_gene_variant	MODIFIER	YPT7
upstream_gene_variant	MODIFIER	CDC5
missense_variant	MODERATI	MIX17
upstream_gene_variant	MODIFIER	AIM34
upstream_gene_variant	MODIFIER	MVP1
upstream_gene_variant	MODIFIER	TAF4
downstream_gene_variant	MODIFIER	TAP42
upstream_gene_variant	MODIFIER	FAR8
downstream_gene_variant	MODIFIER	RSF1
missense_variant	MODERATI	EIS1

upstream_gene_variant	MODIFIER	HOF1
upstream_gene_variant	MODIFIER	ARP9
downstream_gene_variant	MODIFIER	MOT3
upstream_gene_variant	MODIFIER	TVP18
downstream_gene_variant	MODIFIER	ABF2
upstream_gene_variant	MODIFIER	IRC21
upstream_gene_variant	MODIFIER	SDD2
upstream_gene_variant	MODIFIER	RCO1
downstream_gene_variant	MODIFIER	PDS5
downstream_gene_variant	MODIFIER	SCJ1
downstream_gene_variant	MODIFIER	GAS3
upstream_gene_variant	MODIFIER	SKY1
upstream_gene_variant	MODIFIER	GUA1
downstream_gene_variant	MODIFIER	TRS130
upstream_gene_variant	MODIFIER	RKR1
upstream_gene_variant	MODIFIER	GAD1
upstream_gene_variant	MODIFIER	HOR7
upstream_gene_variant	MODIFIER	GTO3
upstream_gene_variant	MODIFIER	RCE1
upstream_gene_variant	MODIFIER	BUL1
synonymous_variant	LOW	DSK2
upstream_gene_variant	MODIFIER	FCP1
upstream_gene_variant	MODIFIER	PRM15
upstream_gene_variant	MODIFIER	SWT21
upstream_gene_variant	MODIFIER	KAR1
upstream_gene_variant	MODIFIER	SRP1
downstream_gene_variant	MODIFIER	DUG3
downstream_gene_variant	MODIFIER	CHS1
downstream_gene_variant	MODIFIER	DGR1
downstream_gene_variant	MODIFIER	CPT1
upstream_gene_variant	MODIFIER	TOM22
missense_variant	MODERATI	KRE33
upstream_gene_variant	MODIFIER	FYV6
upstream_gene_variant	MODIFIER	FPR1
downstream_gene_variant	MODIFIER	EAF7
downstream_gene_variant	MODIFIER	NOP15
downstream_gene_variant	MODIFIER	CYB5
intron_variant	MODIFIER	DBP2
downstream_gene_variant	MODIFIER	RPC19
downstream_gene_variant	MODIFIER	MLF3
upstream_gene_variant	MODIFIER	IMP4
upstream_gene_variant	MODIFIER	MKS1
missense_variant	MODERATI	APJ1
downstream_gene_variant	MODIFIER	NIS1
upstream_gene_variant	MODIFIER	TPM1
upstream_gene_variant	MODIFIER	EOS1

upstream_gene_variant	MODIFIER	DOM34
downstream_gene_variant	MODIFIER	RLP7
downstream_gene_variant	MODIFIER	PET8
missense_variant	MODERATI	HRB1
upstream_gene_variant	MODIFIER	MRP7
downstream_gene_variant	MODIFIER	LST8
upstream_gene_variant	MODIFIER	SIS1
downstream_gene_variant	MODIFIER	MDH2
upstream_gene_variant	MODIFIER	RPL25
downstream_gene_variant	MODIFIER	YGK3
upstream_gene_variant	MODIFIER	VPS68
downstream_gene_variant	MODIFIER	ALR1
downstream_gene_variant	MODIFIER	GAS4
downstream_gene_variant	MODIFIER	DUF1
downstream_gene_variant	MODIFIER	MPD2
missense_variant	MODERATI	HAL9
downstream_gene_variant	MODIFIER	MSH2
upstream_gene_variant	MODIFIER	HMS1
upstream_gene_variant	MODIFIER	EXO1
missense_variant	MODERATI	AKR2
downstream_gene_variant	MODIFIER	SHE4
upstream_gene_variant	MODIFIER	PFY1
upstream_gene_variant	MODIFIER	LEO1
synonymous_variant	LOW	UBP2
downstream_gene_variant	MODIFIER	CAT5
downstream_gene_variant	MODIFIER	IAH1
upstream_gene_variant	MODIFIER	RGA1
upstream_gene_variant	MODIFIER	ATG40
synonymous_variant	LOW	PDR5
upstream_gene_variant	MODIFIER	SLP1
downstream_gene_variant	MODIFIER	ISN1
upstream_gene_variant	MODIFIER	CBC2
upstream_gene_variant	MODIFIER	PPQ1
missense_variant	MODERATI	TCO89
downstream_gene_variant	MODIFIER	CTI6
upstream_gene_variant	MODIFIER	RTT10
downstream_gene_variant	MODIFIER	RLM1
downstream_gene_variant	MODIFIER	RPS6A
upstream_gene_variant	MODIFIER	GLR1
downstream_gene_variant	MODIFIER	SSU1
downstream_gene_variant	MODIFIER	NOG1
upstream_gene_variant	MODIFIER	SEC62
upstream_gene_variant	MODIFIER	RAD1
downstream_gene_variant	MODIFIER	MET12
upstream_gene_variant	MODIFIER	RMI1
synonymous_variant	LOW	SKS1

downstream_gene_variant	MODIFIER	SMA1
downstream_gene_variant	MODIFIER	ERG10
downstream_gene_variant	MODIFIER	SUV3
